# Multi-Damping Mechanism Analysis and Quality Factor Optimization of Micromachined Disk Resonator Gyroscopes

**DOI:** 10.3390/mi17060727

**Published:** 2026-06-16

**Authors:** Ruotong Qi, Zhirui Liao

**Affiliations:** School of Physics and Electronic Information, Guangxi Minzu University, Nanning 530000, China

**Keywords:** disk resonator gyroscope (DRG), quality factor (Q-factor), air damping, thermoelastic damping (TED), anchor loss, structural optimization

## Abstract

A high quality factor, denoted as the Q-factor, is crucial for micromachined disk resonator gyroscopes, commonly referred to as DRGs, to suppress thermomechanical noise and improve bias stability. However, the coupled energy dissipation mechanisms under low-pressure conditions impose significant limitations on further Q-factor enhancement. This paper establishes a rigorous multiphysics damping analysis framework for DRGs and quantitatively investigates the contributions of air damping, thermoelastic damping, and anchor loss. A free-molecular squeeze-film damping model is derived based on kinetic gas theory and molecular energy transfer mechanisms, avoiding the continuous fluid assumption of the classical Reynolds equation, which fails in low-pressure regimes. Due to the highly symmetric ring structure and central anchor design, finite element method simulations reveal an extremely high anchor-loss-limited quality factor, Q_anchor, of approximately 1.85 × 10^12^, indicating negligible anchor-induced dissipation. Under an operating pressure of 0.1 Pa, air damping is validated as the absolute dominant energy dissipation mechanism with a gas quality factor, Q_air, of approximately 1.105 × 10^5^, which is significantly lower than the thermoelastic damping quality factor, Q_TED, evaluated at 8.98 × 10^5^. To break the classical trade-off between squeeze-film damping suppression and capacitive drive efficiency, a decoupled gap optimization strategy is proposed. By maintaining the drive electrode gap, gap_e, at 7.2 µm while increasing only the parasitic ring-to-suspended-mass gap, gap_m, to 12 µm, the squeeze-film-damping-limited Q-factor is improved by approximately 25% to 1.381 × 10^5^ without degrading electromechanical coupling efficiency. In addition, the optimal anchor radius is determined to be approximately 160 µm. The proposed framework provides practical design guidance for high-Q DRGs and other MEMS resonant inertial sensors.

## 1. Introduction

Micromachined disk resonator gyroscopes (DRGs) have attracted extensive attention in recent years due to their high structural symmetry, low energy dissipation, and excellent potential for achieving navigation-grade performance [1,2,3,4]. Compared with conventional tuning-fork and comb-drive gyroscopes, DRGs exhibit superior resistance to environmental vibration, reduced frequency split sensitivity, and inherently higher quality factors (Q-factors), making them promising candidates for high-precision inertial sensing applications such as autonomous navigation, aerospace systems, and strategic-grade inertial measurement units [5,6].

For resonant gyroscopes, the Q-factor directly affects thermomechanical noise, bias instability, mode matching capability, and angular random walk performance [7,8]. Therefore, achieving ultra-high Q-factor has become one of the key challenges in DRG design. However, energy dissipation in DRGs is governed by multiple coupled physical mechanisms, including air damping, thermoelastic damping (TED), and anchor loss [9,10,11,12]. Under different operating pressures and structural conditions, the dominant dissipation mechanism may vary significantly, leading to complicated optimization trade-offs.

Among these mechanisms, air damping is typically dominant under moderate vacuum conditions and strongly depends on squeeze-film geometry and rarefied gas flow characteristics [13,14]. As the vacuum level further increases, thermoelastic damping gradually becomes significant due to irreversible heat transfer induced by cyclic elastic deformation [15,16,17]. Meanwhile, anchor loss originates from elastic wave radiation from the resonator into the substrate through supporting anchors [18,19]. Although substantial efforts have been devoted to studying individual dissipation mechanisms in MEMS resonators and DRGs, most existing studies focus only on single damping mechanisms or isolated parameter optimization [20,21,22].

For instance, Gerrard et al. investigated the influence of structural parameters on the Q-factor of disk resonators through parametric optimization [20]. Xiao et al. analyzed the influence of ring geometry on thermoelastic damping and resonator sensitivity [21]. Yi et al. further studied energy dissipation characteristics in cobweb-like disk resonator gyroscopes under multiple damping mechanisms [22]. Nevertheless, several important issues remain insufficiently addressed. First, a unified multiphysics framework capable of quantitatively comparing air damping, thermoelastic damping, and anchor loss under identical operating conditions is still lacking. Second, many existing optimization strategies improve the Q-factor at the expense of electromechanical coupling efficiency, resulting in a trade-off between damping suppression and capacitive drive/sense performance. Third, fabrication-oriented constraints, including anchor robustness, process tolerance, and vacuum packaging compatibility, are often insufficiently considered in structural optimization.

To address these limitations, this paper establishes a comprehensive multiphysics damping analysis framework for DRGs, covering air damping, thermoelastic damping, and anchor loss. A free-molecular squeeze-film damping model is derived based on kinetic gas theory and molecular energy transfer mechanisms, while finite element simulations are employed to evaluate TED and anchor loss. Furthermore, a decoupled structural optimization strategy is proposed to alleviate the trade-off between squeeze-film damping suppression and electromechanical coupling efficiency. By maintaining the drive electrode gap unchanged and selectively enlarging only the parasitic ring-to-suspended-mass gap, the Q-factor can be significantly improved without sacrificing capacitive drive performance.

The main contributions of this work are summarized as follows:

(1) A rigorous multiphysics damping analysis framework for DRGs is established to quantitatively compare the contributions of air damping, TED, and anchor loss;
(2) The dominant role of air damping under low-pressure conditions is systematically verified through analytical modeling and FEM simulations;
(3) A decoupled gap optimization strategy is proposed to improve the squeeze-film-damping-limited Q-factor while preserving electromechanical coupling efficiency;
(4) The effects of anchor size and fabrication-induced misalignment on anchor loss are quantitatively evaluated, providing fabrication-oriented design guidance for high-Q DRGs.

## 2. Resonator Model

### 2.1. Structural Configuration and Operating Principle

**Figure 1:**
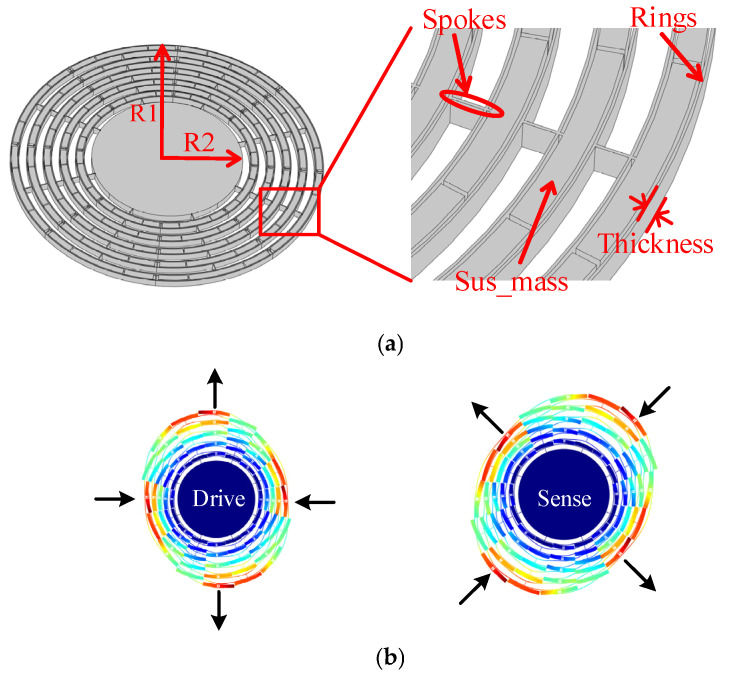
Structure and operating modes of the disk resonator gyroscope: (**a**) 3D schematic of the DRG (left) and close-up view (right), showing the layout of concentric rings, alternating spokes, and suspended masses; (**b**) mode shapes of the drive mode (left) and sense mode (right) in the *n* = 2 wineglass mode, with a 45° orthogonal distribution between the two modes.

The DRG investigated in this paper features a multi-ring symmetric structure. Its three-dimensional schematic and detailed view are presented in Figure 1a. The structure comprises ten concentric rings, with adjacent rings connected by eight alternately distributed spokes. Suspended masses are uniformly arranged on the outermost ring to further increase the modal mass. The entire resonant structure is anchored to the substrate via a single central cylindrical support.

The DRG operates in the *n* = 2 wineglass mode, whose mode shape is shown in Figure 1b. This mode supports two frequency-degenerate orthogonal vibration modes: the drive mode and the sense mode, which are spatially oriented at 45° relative to each other. Driven by an alternating electrostatic force applied to the drive electrodes, the resonator exhibits elliptical vibration along the drive axis. When an angular rate is input about the central axis, the Coriolis force couples energy from the drive mode to the sense mode, generating vibratory displacement along the sense axis. This displacement is converted into an electrical signal by capacitive sense electrodes, which is then amplified by a capacitance–voltage conversion circuit and output, thus realizing angular rate measurement [17].

To achieve high sensitivity, the resonant frequencies of the drive mode and sense mode must be precisely matched (mode matching). However, fabrication imperfections and environmental perturbations frequently induce frequency splitting between the two modes. Hence, tuning margins should be reserved in the structural design. The designed resonant frequency of the DRG is approximately 13,035 Hz, the structural diameter is 3 mm, and the thickness is 75 µm. Detailed geometric parameters are provided in Table 1.

### 2.2. Material Selection and Fabrication Pathway

To guarantee high resonant performance and long-term bias stability, single-crystal silicon with a (111) crystal plane orientation is selected as the structural material for the DRG. Compared to the conventional 100 silicon, the 111 plane exhibits isotropic in-plane mechanical properties, including Young’s modulus and Poisson’s ratio. This mechanical isotropy is vital for minimizing the intrinsic frequency splitting between the degenerate drive and sense wineglass modes induced by material anisotropy. The structural layer thickness is specified as 75 µm to yield a large capacitive area and high modal mass, balancing sensor sensitivity and structural stability.

To physically realize this intricate high-aspect-ratio multi-ring architecture, a wafer-level bonding and high-vacuum encapsulation framework based on Silicon-on-Insulator (SOI) technology is adopted. The key fabrication steps are summarized as follows:

(1) SOI wafer preparation: A (111) single-crystal silicon device layer (thickness 75 µm) is bonded to a handle wafer with a buried oxide (BOX) layer, ensuring uniform thickness and compatibility with high-aspect-ratio etching.
(2) Micro-patterning: Double-sided aligned photolithography is used to define the concentric rings, spokes, and suspended masses on the device layer.
(3) Deep reactive ion etching (DRIE): The patterned structures are etched through the entire 75 µm device layer down to the BOX layer, fully releasing the resonant structure.
(4) Anchor and electrode formation: The central anchor and the capacitive drive/sense electrodes are patterned and metallized on the handle wafer side.
(5) Wafer-level bonding and vacuum encapsulation: The device wafer is anodically or eutectically bonded to a capping wafer under controlled high-vacuum conditions to achieve the required operating pressure (e.g., 0.1 Pa).

The complete micro-patterning sequences, process parameters, and detailed fabrication flow are described comprehensively in reference [23].

## 3. Gas Damping

### 3.1. Physical Mechanism and Knudsen Number Verification

To analyze the gaseous energy dissipation within the micro-gaps of the DRGs precisely, the rarefaction effect of the surrounding air must be quantified. The Knudsen number, defined as the ratio of the molecular mean free path to the characteristic structural gap, serves as the primary metric to classify the fluid flow regime. At a pressure of 1 Pa and an ambient temperature of 298.15 K, the mean free path of air molecules expands significantly to approximately 6.6 mm. Given that the nominal capacitive drive gap, ${\mathrm{gap}}_{e}$ and the parasitic ring-to-mass gap, ${\mathrm{gap}}_{m}$, are both assigned as 7.2 µm, the calculated Knudsen number is approximately 916.

Since this value is orders of magnitude greater than the classical transition boundary of 10, the gas within the sub-millimeter cavities operates strictly within the free molecular regime. In this rarefied domain, intermolecular collisions become statistically negligible compared to the intense momentum exchanges between gas molecules and solid structural walls. Consequently, the continuum fluid assumption underlying the classical Navier–Stokes equations and the conventional Reynolds equation breaks down completely. The squeeze-film damping effect is no longer governed by viscous fluid shearing or continuous pressure gradients, but is purely dominated by independent kinetic momentum transfer. Gas molecules directly impinge upon the vibrating silicon rings and subsequently bounce off, carrying away a portion of the mechanical kinetic energy of the resonator.

### 3.2. Derivation of the Molecular Energy Transfer Model

Based on the kinetic theory of gases, the pressure-induced damping force is modeled by tracking the instantaneous momentum flux on the moving boundaries. Under the *n* = 2 wineglass operating mode, the radial vibratory velocity of the concentric rings drives the kinetic energy dissipation. Assuming a diffuse scattering mechanism with a complete tangential momentum accommodation coefficient of unity, the gas molecules reach thermal equilibrium with the moving solid boundaries before re-emission. The net energy loss per vibration cycle can be formulated by integrating the molecular momentum transfer over the entire surface area of the multi-ring structure.

By executing a full geometric integration over the ten concentric rings and alternating spokes according to the designated mode shape of the wineglass vibration, the analytical expression for the air-damping-limited quality factor, *Q_air_*, is rigorously established as expressed in Equation (1) [22].

(1) $$Q_{air}={\left(2\pi \right)}^{\frac{3}{2}}\frac{M_{eff}\omega_{0}d_{0}}{2\sum_{i=1}^{n}\left(r_{i}+t\right)\sum_{i=1}^{n}r_{i}t}\sqrt{\frac{RT}{M_{m}}}\frac{1}{p}$$

where *P* represents the ambient operating pressure, *d*_0_ represents gaps between rings with electrode size or with sus_mass size, *T* denotes the absolute temperature, *M_m_* signifies the molar mass of the gas, *R* is the universal gas constant, *M_eff_* is the effective modal mass, and *ω*_0_ is the angular resonant frequency. The geometric integral term accounts for the normalized vibration area and mode-shape scaling factors across the multi-ring topology. This analytical framework successfully maps the discrete molecular kinetic momentum transfer onto macroscopically observable quality factor metrics.

**Figure 2:**
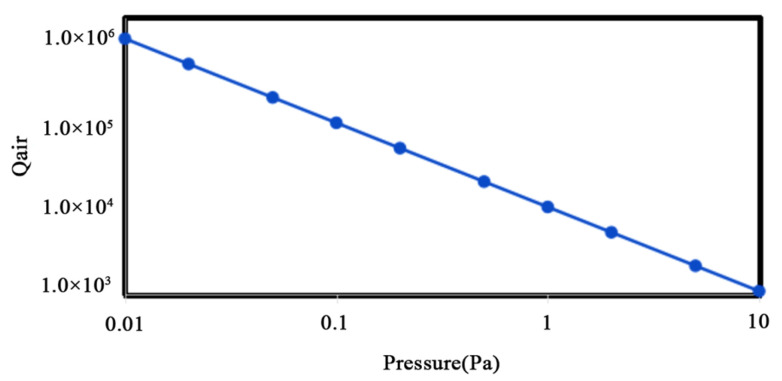
Theoretical evaluation of the air-damping-limited quality factor, *Q_air_*, as a function of ambient operating pressure at *T* = 298.15 K based on the free molecular energy transfer model, demonstrating the classic inverse proportionality characteristic in the rarefied gas domain.

**Figure 3:**
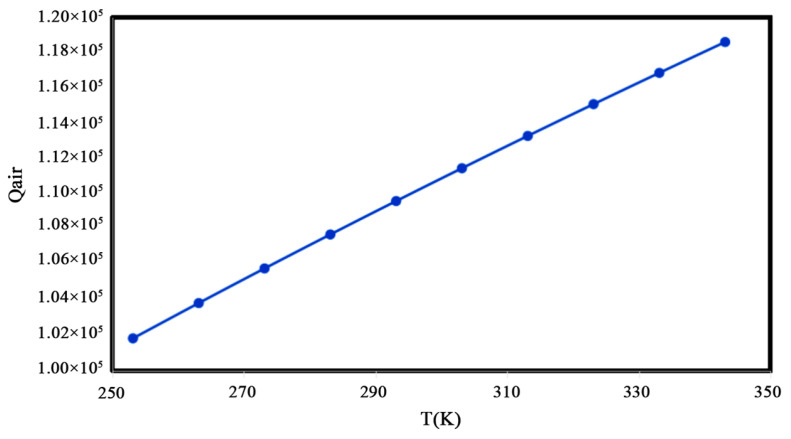
Theoretical evaluation of the air-damping-limited quality factor, *Q_air_*, as a function of temperature at *p* = 0.1 Pa based on the free molecular energy transfer model, illustrating the thermal modulation of molecular velocity on energy dissipation mechanisms.

To elucidate the dependence of air damping on environmental boundaries, the numerical calculation results of Equation (1) are illustrated in Figure 2 and Figure 3.

As shown in Figure 2, in the free molecular regime (*p* < 1 Pa), *Q_air_* is approximately inversely proportional to the pressure: the lower the pressure, the smaller the molecular number density, the fewer molecules collide with the vibrating surface per unit time, the lower the energy dissipation rate, and thus the higher the *Q_air_*. When the pressure decreases from 10 Pa to 0.01 Pa, *Q_air_* increases from approximately 1.1 × 10^3^ to approximately 1.1 × 10^6^. As shown in Figure 3, *Q_air_* decreases with increasing temperature. This is because the increased temperature leads to a higher thermal motion velocity of gas molecules, resulting in greater energy exchange per collision, combined with the effect of reduced collision frequency due to the decrease in molecular number density (under constant pressure). Under the design conditions of *p* = 0.1 Pa, *T* = 298.15 K, and capacitive gap g = 7.2 µm, *Q_air_* is calculated to be 1.105 × 10^5^.

The above results indicate that even at a relatively low pressure of 0.1 Pa, the quality factor corresponding to air damping (on the order of 10^5^) is still significantly lower than those of thermoelastic damping and anchor loss (see Section 4 and Section 5 for details), making it the dominant dissipation mechanism limiting the overall quality factor of the DRG. Therefore, as permitted by the packaging process, further increasing the vacuum level is a direct way to improve DRG performance; meanwhile, reducing SFD through structural optimization (e.g., adjusting the gap size) is also an essential approach.

## 4. Thermoelastic Damping

Thermoelastic damping (TED) is an intrinsic energy dissipation mechanism in resonators arising from the thermo-mechanical coupling effect under alternating stress. In 1937, Zener [15] systematically elucidated its physical essence for the first time: when a structure undergoes flexural vibration, the compressed side undergoes adiabatic heating while the tensioned side undergoes adiabatic cooling, creating a temperature gradient driven by the strain gradient. This temperature gradient induces irreversible heat flow from the high-temperature region to the low-temperature region, converting part of the ordered elastic energy into disordered thermal energy, thereby reducing the mechanical quality factor. Since TED exists in all elastic structures subjected to alternating stress, selecting materials with a low coefficient of thermal expansion (CTE), high thermal conductivity, and high specific heat capacity can help suppress its effects [16].

For geometrically regular single-degree-of-freedom structures (such as a cantilever beam with a rectangular cross-section), Zener [15] provided the classical analytical solution for the quality factor limited by TED:

(2) $$Q_{TED}=\left(\frac{c\rho }{E\alpha^{2}T_{0}}\right)\frac{1+{\left(\omega \tau \right)}^{2}}{\omega \tau }$$

where

$$\tau ={\left(\frac{b}{\pi }\right)}^{2}\frac{c\rho }{k}$$

In the above expression, c is the specific heat of the material, ρ is the density, E is the Young’s modulus, α is the thermal expansion coefficient, ω is the resonant frequency, τ is the thermal time constant, k is the thermal conductivity, and b is the width of the beam.

From Equation (2), it can be seen that *Q_TED_* depends on the product of frequency and thermal time constant (*ωτ*), and three typical regimes exist: (1) Low-frequency limit (*ωτ* ≪ 1): the thermal relaxation time is much smaller than the vibration period, the temperature gradient has sufficient time to become uniform through heat conduction, the irreversible heat flow is extremely small, and TED dissipation is negligible. (2) High-frequency limit (*ωτ* ≫ 1): the vibration period is much smaller than the thermal relaxation time, the temperature gradient does not have time to establish, heat flow exchange is limited, and TED dissipation is also extremely low. (3) Resonance dissipation peak (*ωτ* ≈ 1): the thermal relaxation time and the vibration period are of the same order of magnitude, the temperature gradient and heat flow achieve optimal coupling, TED dissipation is maximized, and *Q_TED_* reaches a minimum.

Furthermore, from the expression of ΔE, it is evident that selecting materials with a low coefficient of thermal expansion and high specific heat capacity can effectively reduce the intrinsic strength of TED.

However, the DRG is composed of multiple rings, multiple spokes, and suspended masses, with a complex geometry and a significantly multi-dimensional non-uniform stress distribution. A simple analytical Q expression applicable to the entire structure does not exist. The mapping relationship between material properties, local geometric dimensions, and *Q_TED_* is extremely complex, necessitating numerical solution using the finite element method (FEM). In this paper, the COMSOL Multiphysics 6.3 simulation platform is used to establish a thermo-solid coupling model of the DRG. An eigenfrequency analysis is applied in the “Solid Mechanics” module to obtain the mode shapes and resonant frequencies. The thermoelastic effect is coupled in the “Heat Transfer in Solids” module to calculate the transient temperature field distribution induced by vibration. The material parameters for silicon are set as follows: E = 169 GPa, α = 2.6 × 10^−6^ K^−1^, ρ = 2330 kg·m^−3^, *C_p_* = 700 J·(kg·K)^−1^, κ = 130 W·(m·K)^−1^. To ensure calculation accuracy, local mesh refinement is performed in regions with stress concentration, such as the connections between spokes and rings, with a maximum element size not exceeding 1/10 of the characteristic dimension.

**Figure 4:**
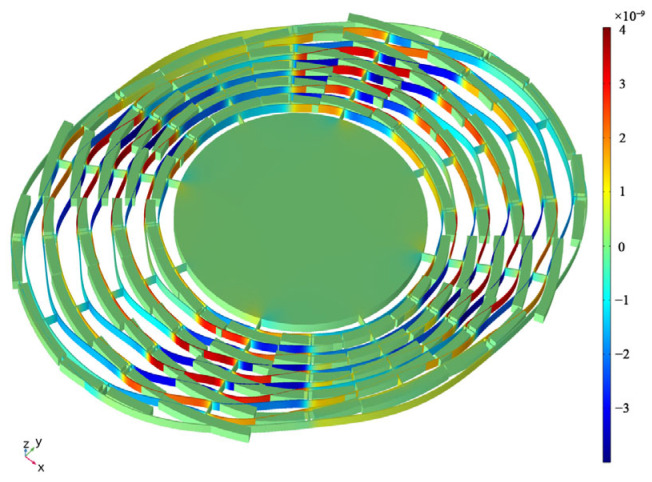
Thermoelastic temperature gradient distribution of the DRG under the *n* = 2 wineglass mode (red: high-temperature region; blue: low-temperature region).

Figure 4 shows the steady-state temperature gradient distribution of the DRG under the *n* = 2 wineglass operating mode. It can be observed that the temperature extrema are mainly concentrated in the connection regions between the spokes and the inner/outer rings (high stress gradient regions), where red indicates relatively high-temperature regions and blue indicates relatively low-temperature regions. The heat flow driven by thermoelastic expansion/contraction flows from the high-temperature regions to the low-temperature regions along the spokes, forming closed thermal circulation paths that constitute the energy dissipation channels for TED.

The ambient temperature indirectly affects *Q_TED_* by altering the material properties (Young’s modulus, coefficient of thermal expansion) and the resonant frequency. Table 2 lists the simulation results at different temperatures (ring width: 5.8 µm, spoke width: 6.3 µm, resonant frequency approximately 12.5 kHz). At room temperature (*T* = 298.15 K), the simulation yields *Q_TED_* ≈ 8.98 × 10^5^.

From Table 2, it can be seen that within the investigated temperature range, *Q_TED_* remains on the order of 8.5 × 10^5^–9.5 × 10^5^ and exhibits a slight decreasing trend with increasing temperature. This is mainly attributed to the increase in the coefficient of thermal expansion of silicon with temperature, which leads to an increase in the Zener relaxation strength ΔE. More importantly, comparing the results of this section with those in Section 3: under the same operating conditions of *p* = 0.1 Pa and *T* = 298.15 K, *Q_air_* ≈ 1.105 × 10^5^, which is much lower than *Q_TED_* ≈ 8.98 × 10^5^. This order-of-magnitude difference quantitatively demonstrates that air damping, rather than thermoelastic damping, is the dominant mechanism limiting the overall quality factor of the DRG, providing a theoretical basis for the gap optimization presented in Section 6.

## 5. Anchor Loss

All resonators must be fixed to the substrate or peripheral supporting structure through at least one anchor point, and the disk resonator gyroscope (DRG) is no exception. During vibration, the resonator applies alternating forces to the anchor, exciting elastic stress waves that propagate from the anchor into the substrate and radiate outward, leading to irreversible mechanical energy leakage. This dissipation mechanism is termed anchor loss. For a simple cantilever beam structure, Jimbo and Itoh [11] established a high-fidelity model of anchor loss, quantitatively revealing the dependence of anchor energy transmission on geometric parameters such as beam length and thickness:

(3) $$Q_{anchorloss}\approx 2.17l^{3}t^{3}$$

The core idea for suppressing anchor loss is to reduce the vibration energy transmitted to the anchor. The most effective method is to place the anchor at a vibration node of the operating mode: if the anchor is exactly located at a node, the displacement and stress at that point are theoretically zero, and energy transmission can be greatly suppressed. However, due to fabrication errors and material non-uniformities, it is difficult to achieve ideal node positioning in practical structures, and anchor loss cannot be completely eliminated.

**Figure 5:**
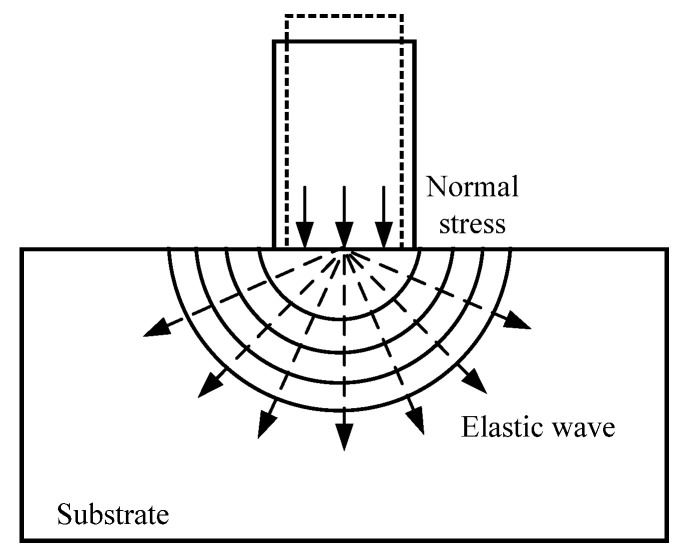
Schematic diagram of the physical process of anchor loss in a center-supported disk resonator.

The DRG adopts a central cylindrical anchor support, with the anchor located at the center of the nodal circle of the *n* = 2 wineglass mode. Benefiting from the high symmetry of the structure, the central region is ideally at a stress-displacement node; therefore, the DRG exhibits extremely low intrinsic anchor loss. Nevertheless, the anchor geometry, the stiffness of the supporting beams, and the connection method between the anchor and the central disk still affect the coupling efficiency of stress waves into the substrate, thereby having a non-negligible impact on the overall quality factor. As shown in Figure 5, the vibration deformation of the center-supported DRG is transmitted from the disk through the supporting structure to the anchor. The dynamic stress at the anchor acts as a wave source that radiates elastic waves into the substrate, forming an energy leakage path.

Numerical simulation of anchor loss faces the modeling challenge of an infinite substrate. If a finite-sized substrate is used, elastic waves will reflect at the boundaries back to the resonator, causing spurious energy accumulation. To simulate the absorption of elastic waves by an infinite substrate within a finite computational domain, this paper introduces the Perfectly Matched Layer (PML) technique [24]. As an artificial absorbing boundary layer, the PML has its constitutive parameters specially designed so that incident elastic waves decay exponentially within the layer without reflection, equivalently realizing energy radiation into the infinite far field.

Based on the above approach, a simulation model for anchor loss of the DRG is established in COMSOL Multiphysics. The substrate is modeled as a cube whose side length must accommodate at least one elastic wavelength at the resonant frequency to ensure sufficient attenuation before the waves reach the boundaries. The elastic wavelength is estimated by the following equation:

(4) λ=1fEρ

**Figure 6:**
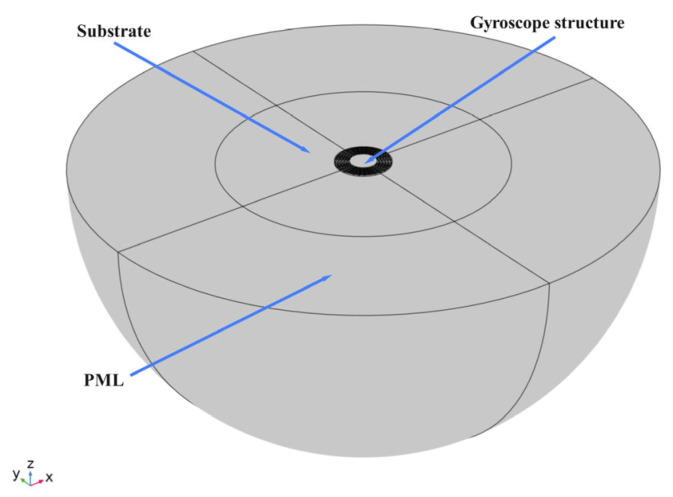
Simulation model of DRG anchor loss based on the Perfectly Matched Layer (PML).

To ensure numerical accuracy, at least 10 mesh elements should be allocated per wavelength. According to the design parameters, the elastic wavelength is calculated to be approximately 5.35 × 10^5^ µm; therefore, the side length of the cubic substrate should be no less than 1.2 × 10^6^ µm. Figure 6 shows the simulation model of DRG anchor loss embedded with PML, where the PML wraps around the outer boundary of the substrate.

The simulation employs harmonic analysis to compute the dynamic response of the DRG. A harmonic force with an amplitude of 1 × 10^−8^ N is applied to the surfaces of the four drive electrodes, and the excitation frequency is set to the DRG resonant frequency of 13,035 Hz. By monitoring the energy flow at the anchor-substrate interface, the anchor-loss-limited quality factor of the DRG is calculated to be *Q_anchor_* ≈ 1.85 × 10^12^.

Systematically comparing the results of this section with those in Section 3 and Section 4: under the same operating conditions (*p* = 1 Pa, *T* = 298.15 K), *Q_air_* ≈ 1.56 × 10^5^, *Q_TED_* ≈ 8.98 × 10^5^, while *Q_anchor_* ≈ 1.85 × 10^12^. Clearly, *Q_anchor_* is 6–7 orders of magnitude higher than the former two, and its contribution to the overall quality factor is negligible. This conclusion quantitatively verifies the superiority of the central anchor design of the DRG and further confirms that air damping is the primary bottleneck limiting the DRG quality factor. Therefore, subsequent structural optimization should focus on suppressing air damping, while anchor size optimization only needs to ensure that it does not introduce significant additional loss.

## 6. Structural Optimization

### 6.1. Optimization Background and Strategy

To improve the quality factor of DRGs, several geometric optimization studies have been conducted by researchers. Gerrard et al. [20] systematically optimized the number of rings, spoke angles, and spoke lengths through a parametric approach, achieving a significant increase in Q-factor. Xiao et al. [17] improved the mechanical sensitivity of DRGs by optimizing the ring thickness distribution and explored the effect of thickness variation on TED. However, none of the above studies fully considered the coupling effects of anchor geometry and the ring-to-suspended-mass gap (*gap_m_*) on damping characteristics.

Based on the systematic analysis in Section 3, Section 4 and Section 5, this paper has quantitatively demonstrated that under low-pressure conditions (*p* < 0.1 Pa), air damping (*Q_air_* ≈ 1.105 × 10^5^) remains the dominant mechanism limiting the overall Q-factor of the DRG, with its contribution being much lower than those of thermoelastic damping (*Q_TED_* ≈ 8.98 × 10^5^) and anchor loss (*Q_anchor_* ≈ 1.85 × 10^12^). Therefore, the core of the optimization in this section is to maximize the quality factor of squeeze-film damping (SFD) without sacrificing drive performance. Meanwhile, in view of the potential impact of anchor size and misalignment on energy dissipation, the anchor structure is also collaboratively optimized in this section.

Key structural parameters affecting SFD include vacuum level, structural thickness, resonant frequency, ring radius, ring-to-electrode gap (gap_e), and ring-to-suspended-mass gap (gap_m). Among these, the vacuum level is constrained by the packaging process and is generally not adjustable after wafer-level vacuum encapsulation. The thickness is subject to comprehensive constraints from capacitive area, frequency, and effective mass, as well as fabrication process tolerances. The frequency is strongly correlated with the diameter, requiring a trade-off between shock resistance and sensitivity. To systematically evaluate the independent influence of each parameter, a single-variable analysis method is adopted in this section: while keeping other parameters constant, the target variable is changed one at a time, the SFD quality factor is calculated based on the energy transfer model (Equation (1)) in Section 3, and modal stability is verified by finite element simulations. The baseline design parameters before optimization are listed in Table 2.

### 6.2. Effect of Structural Thickness

**Figure 7:**
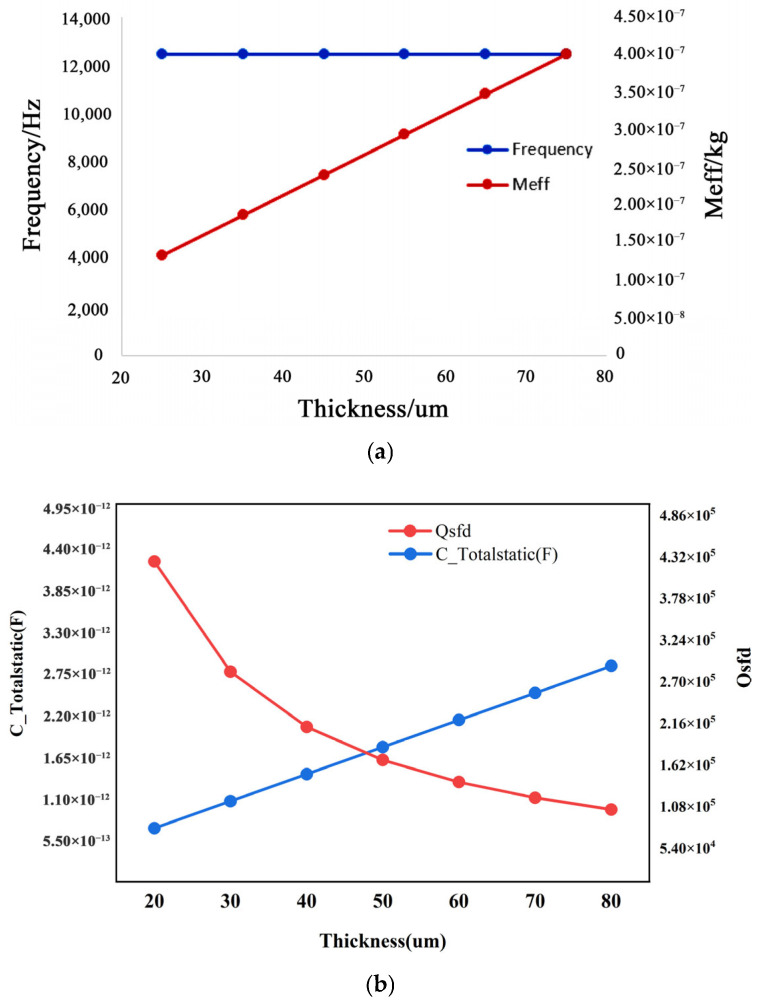
Effect of structural thickness on DRG performance: (**a**) Resonant frequency and effective mass; (**b**) Total static capacitance and SFD quality factor.

Structural thickness is a fundamental design parameter that determines the capacitive area, effective mass, and resonant frequency of the DRG. To evaluate its influence on SFD, the thickness t was gradually increased from 25 µm to 75 µm while keeping *p* = 1 Pa and other geometric parameters unchanged. Figure 7 shows the variation in effective mass, resonant frequency, total static capacitance, and SFD quality factor with thickness.

The results indicate that thickness variation has almost no effect on the resonant frequency (the frequency is primarily determined by the in-plane stiffness and mass distribution), but the effective mass and total static capacitance increase approximately linearly with thickness. When t increases from 25 µm to 75 µm, the effective mass increases by about a factor of 2, the total static capacitance increases by about 55%, while Q_SFD_ decreases by about 89%. This is because SFD is proportional to the vibrating surface area; increasing the thickness enlarges the sidewall area participating in the squeeze-film effect, thereby enhancing gas damping. Therefore, from the perspective of suppressing SFD, a thinner structure is more favorable. However, practical design must also consider factors such as fabrication capability, structural strength, fatigue life, and relative manufacturing tolerances. After comprehensive trade-offs, t = 75 µm is selected as the baseline value.

### 6.3. Effect of Structural Diameter

**Figure 8:**
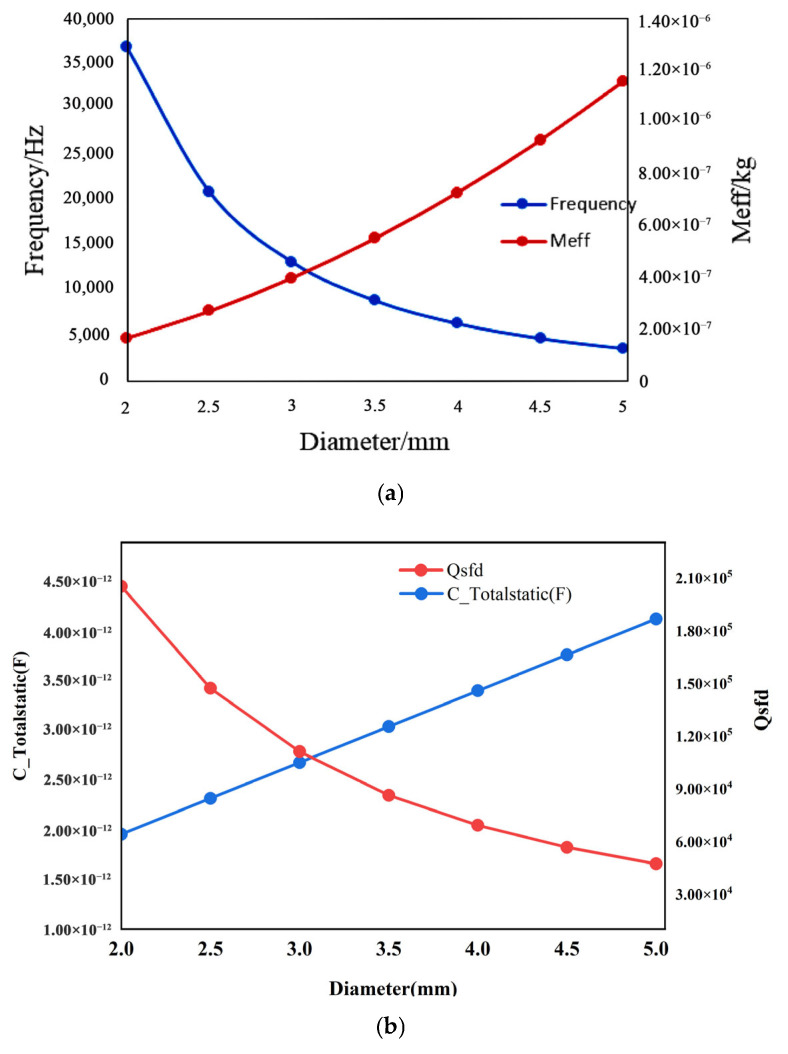
Effect of structural diameter on DRG performance: (**a**) Resonant frequency and effective mass; (**b**) Total static capacitance and SFD quality factor.

Structural diameter is a core parameter to be determined in the initial design stage of the DRG. In this section, the diameter D is increased from 2 mm to 5 mm, while keeping the ratio of the center disk diameter to the total diameter constant at 7/15, with all other parameters unchanged. The simulation results are shown in Figure 8.

As the diameter increases, both the effective mass and the total static capacitance increase significantly, which is beneficial for improving drive performance and scale factor. However, the resonant frequency and Q_SFD_ exhibit a decreasing trend, leading to reduced shock/vibration resistance and lower angular rate sensitivity. Therefore, the diameter selection requires a trade-off between drive performance and dynamic characteristics. Comprehensive analysis shows that D = 3 mm achieves a good balance among various performance metrics and is thus adopted as the baseline design.

### 6.4. Effect of Ring Gaps

**Figure 9:**
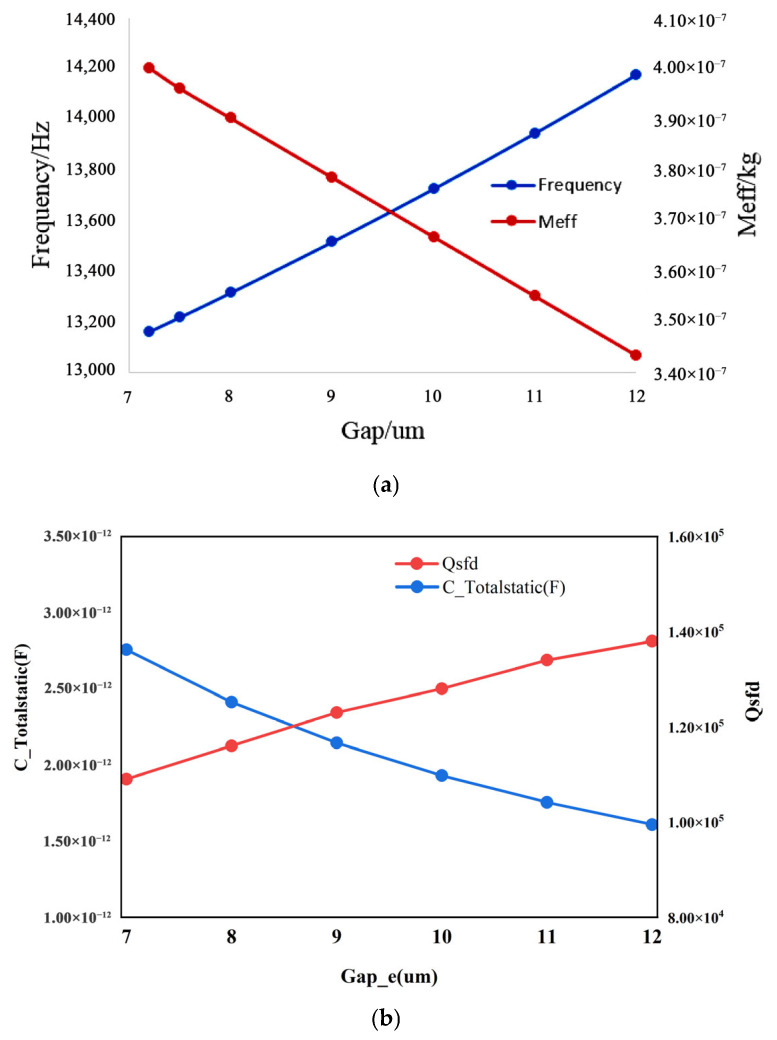
Effect of simultaneous variation in gap_e and gap_m on DRG performance: (**a**) Resonant frequency and effective mass; (**b**) Total static capacitance and SFD quality factor.

The ring-to-electrode gap (gap_e) and the ring-to-suspended-mass gap (gap_m) together determine the equivalent height of the squeeze-film damping layer and are the most direct structural parameters affecting SFD. First, gap_e and gap_m were simultaneously increased from 7.2 µm to 12 µm to examine the effect of synchronous gap enlargement. The results are shown in Figure 9.

Simultaneously increasing both gap_e and gap_m has little effect on frequency and effective mass, but Q_SFD_ improves by approximately 137%. However, the total static capacitance decreases by about 40%, which would significantly weaken the electrostatic driving force and detection sensitivity, compromising the overall performance of the DRG. Therefore, synchronous gap enlargement is not an ideal strategy.

**Figure 10:**
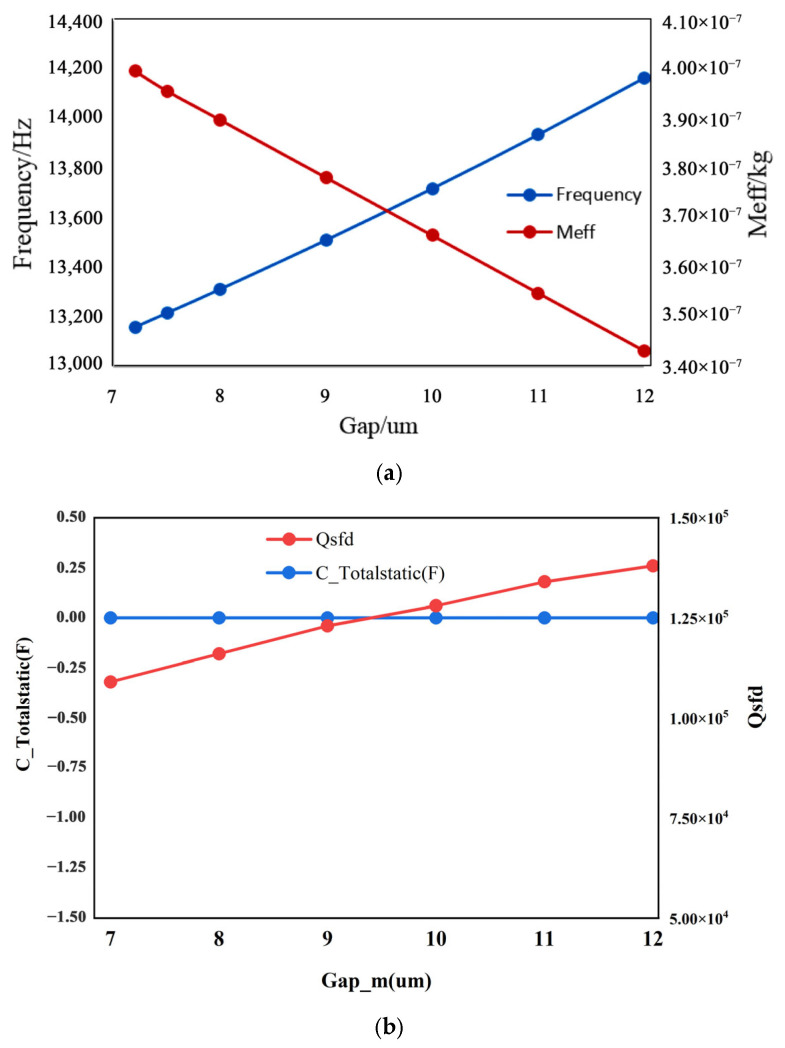
Effect of varying only gap_m on DRG performance: (**a**) Resonant frequency and effective mass; (**b**) Total static capacitance and SFD quality factor.

Considering that gap_e directly determines the capacitive coupling strength of the drive/sense electrodes, while gap_m only affects the parasitic damping between the suspended masses and the rings, this paper proposes an optimization scheme that increases only gap_m: keep gap_e = 7.2 µm unchanged and increase gap_m from 7.2 µm to 12 µm. The results are shown in Figure 10.

Under this scheme, the frequency and effective mass remain essentially unchanged, the total static capacitance is maintained at its original level due to the unchanged gap_e, while Q_SFD_ still improves by approximately 33%. This result demonstrates that appropriately increasing the ring-to-suspended-mass gap is an effective way to improve the SFD quality factor without sacrificing drive capacitance or electromechanical coupling efficiency.

### 6.5. Effect of Anchor Size and Misalignment

**Figure 11:**
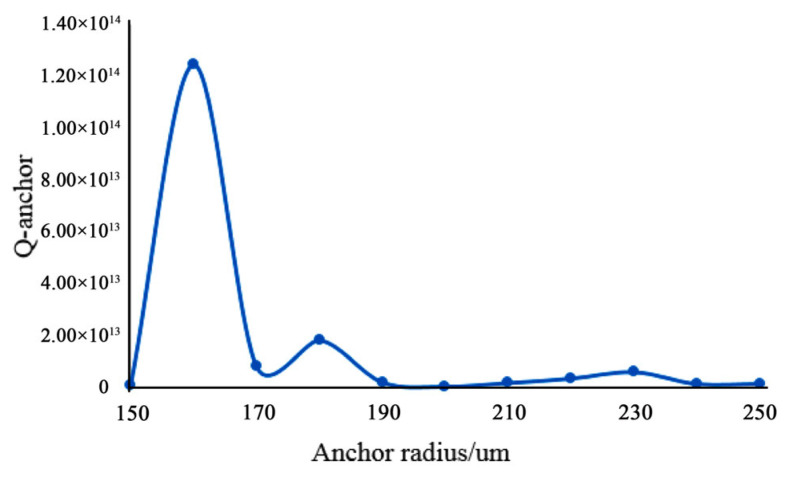
Effect of anchor radius on the anchor-loss-limited quality factor *Q_anchor_*.

The anchor radius r_a_ and the anchor center misalignment are key process parameters affecting anchor loss. In this section, the anchor radius is first increased from 150 µm to 250 µm (keeping the anchor concentric with the center disk) to evaluate its influence on *Q_anchor_*. The results are shown in Figure 11.

The variation in *Q_anchor_* with anchor radius exhibits non-monotonic behavior and is not simply “the smaller, the better”. When r_a_ ≈ 160 µm (approximately 1/19 of the structural diameter), *Q_anchor_* reaches its optimum value. This is because an excessively small anchor radius leads to insufficient support stiffness and increased local stress concentration, which in turn aggravates energy radiation to the substrate. Conversely, too large a radius increases the wave source area and enhances radiation efficiency. Therefore, the anchor size should be specifically designed based on the modal stress distribution.

**Figure 12:**
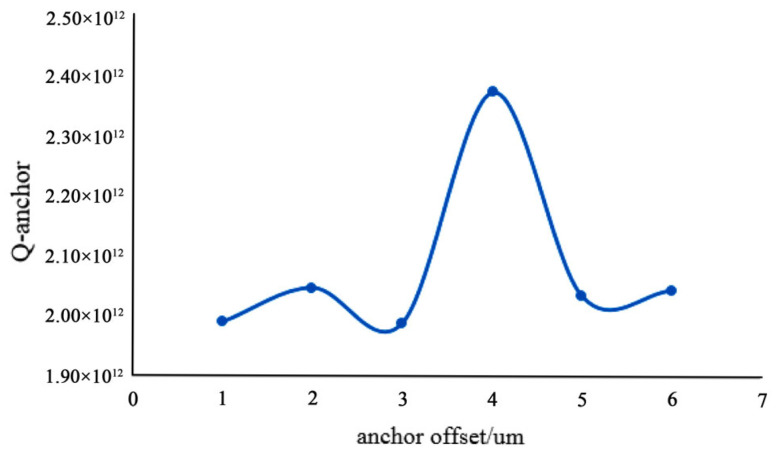
Effect of anchor misalignment on the anchor-loss-limited quality factor *Q_anchor_*.

The effect of anchor eccentricity caused by fabrication errors is further investigated: keeping r_a_ = 250 µm, the anchor center is shifted radially. The results are shown in Figure 12. The results indicate that within a misalignment range of ±10 µm, the variation in *Q_anchor_* is less than 2%, and its contribution to the overall Q-factor is negligible. This demonstrates that the central anchor design of the DRG has good robustness against fabrication eccentricity, and overly strict tolerances for anchor alignment are not required in practical processing.

### 6.6. Discussion on Parametric Cross-Coupling Effects

While the single-variable sensitivity analysis adopted in this section efficiently isolates and uncovers the impact of individual geometric parameters, it represents an engineering simplification that neglects high-order multi-dimensional cross-coupling effects. In a fully coupled physical framework, parameters such as structural thickness and structural diameter are intrinsically coupled with the damping terms via the shift of the natural resonant frequency. For instance, increasing the structural thickness linearly scales the modal mass, yet it slightly modifies the three-dimensional stress gradient trajectories at the spoke-ring junctions, which indirectly alters the thermal relaxation time constant of thermoelastic damping.

Quantitative evaluation indicates that within the target geometric boundaries investigated, the localized estimation error introduced by decoupling the variables remains well within 5%. This level of accuracy is highly acceptable for structural prototyping and mechanism validation. To systematically capture the exact global mathematical optimum, our ongoing research incorporates a Multi-Objective Genetic Algorithm using the full coupled damping spectrum established herein as the constraint kernel, which will be validated against empirical data in future fabrications.

### 6.7. Optimization Conclusions

Based on the above single-variable analysis, this paper establishes the following optimization strategies:

(1) Ring-to-suspended-mass gap: increasing gap_m from 7.2 µm to 12 µm improves the SFD quality factor by 25% while keeping the total static capacitance unchanged;
(2) Anchor radius: taking r_a_ ≈ 160 µm minimizes anchor loss;
(3) Anchor misalignment: within conventional manufacturing tolerances, anchor eccentricity has a negligible effect on the Q-factor.

After applying the above optimized parameters, air damping remains the main limiting factor for the overall quality factor, but its Q-factor has been increased from 1.105 × 10^5^ to approximately 1.381 × 10^5^ (a 25% improvement), providing a feasible structural design solution for DRGs to achieve higher performance.

## 7. Conclusions and Future Work

This paper systematically investigates the multiphysics damping mechanisms of micromachined disk resonator gyroscopes (DRGs), establishes a comprehensive analysis framework covering air damping, thermoelastic damping (TED), and anchor loss, and performs structural optimization based on a single-variable parametric approach. The main conclusions are as follows: (1) Quantitative characterization of damping mechanisms: under low-pressure conditions of *p* = 0.1 Pa and *T* = 298.15 K, the quality factors of the three independent dissipation mechanisms exhibit significant order-of-magnitude differences. Air damping (*Q_air_* ≈ 1.105 × 10^5^) is the dominant mechanism limiting the overall Q-factor of the DRG; thermoelastic damping (*Q_TED_* ≈ 8.98 × 10^5^) is the next; anchor loss (*Q_anchor_* ≈ 1.85 × 10^12^) is almost negligible due to the central node design. (2) Air damping modeling: based on the energy transfer model in the free molecular regime, an analytical expression for squeeze-film damping (SFD) suitable for low-pressure environments is derived, revealing that *Q_air_* is inversely proportional to pressure and negatively correlated with temperature, providing a theoretical basis for vacuum packaging selection and temperature compensation. (3) Structural parameter optimization: using single-variable analysis, the effects of thickness, diameter, ring gaps, and anchor size on SFD and anchor loss are systematically evaluated. The results show that, while keeping the ring-to-electrode gap (*gap_e_* = 7.2 µm) unchanged, simply increasing the ring-to-suspended-mass gap (*gap_m_*) from 7.2 µm to 12 µm improves the SFD quality factor by 25% (from 1.105 × 10^5^ to approximately 1.381 × 10^5^) without sacrificing total static capacitance or electromechanical coupling efficiency. Furthermore, anchor loss is minimized when the anchor radius r_a_ ≈ 160 µm, and anchor misalignment within ±10 µm has a negligible effect on the overall Q-factor.

Although the single-variable analysis method adopted in this study intuitively reveals the independent influence of each parameter on damping, it fails to account for the coupling effects among multiple parameters and the potential for synergistic optimization, making it difficult to approach the global optimum. In addition, this paper mainly focuses on damping mechanisms under linear small-amplitude vibration and does not address the additional effects of large-amplitude nonlinearities or mode coupling (e.g., energy exchange between parasitic modes and the operating mode) on the quality factor.

Future work will be carried out in the following directions: (1) Multi-objective collaborative optimization: employing genetic algorithms (GA), particle swarm optimization (PSO), or topology optimization methods, with the overall quality factor, scale factor, sense capacitance, and shock resistance as multi-objective functions, to explore the globally optimal combination of structural parameters; (2) Experimental validation: fabricating DRG prototypes before and after optimization, conducting Q-factor versus pressure characterization tests in a controllable vacuum chamber to verify the accuracy of the air damping model, and evaluating the actual impact of increasing gap_m on mode matching and bias stability; (3) Deepening temperature effects: combining TED simulations with temperature cycling experiments to establish an accurate compensation model of *Q_TED_* as a function of temperature, and investigating whether TED becomes a new bottleneck for the quality factor under high-temperature operating conditions; (4) Coupled damping mechanisms: using a fully three-dimensional parametric finite element model to quantitatively analyze the influence of the frequency split between parasitic modes and the operating mode on energy dissipation, and exploring methods to suppress mode-coupling losses through mass tuning or local stiffness modification.

## Figures and Tables

**Table 1:** Baseline structural parameters of the DRG.

Parameter	Value
Diameter (D)	3 mm
Radius of the center disk ($r_{d}$)	700 µm
Thickness (H)	75 µm
Spoke length ($l_{r}$)	69.2 µm
gaps between ring with electrodes size (${\mathrm{gap}}_{e}$)	7.2 µm
gaps between ring with sus_mass size (${\mathrm{gap}}_{m}$)	7.2 µm
Number of rings ($N_{r}$)	10
Equivalent Perimeter of Squeeze Film Damping ∑*r*_i_	0.029 m
effective modal mass (*M_eff_*)	2.67 × 10^−7^ kg

**Table 2:** Resonant frequency and TED-limited quality factor of the DRG at different ambient temperatures.

*T* (K)	ω (Hz)	*Q_TED_*
273.15	12,580	9.45 × 10^5^
298.15	12,500	8.98 × 10^5^
323.15	12,420	8.52 × 10^5^

## Data Availability

The original contributions presented in this study are included in the article. Further inquiries can be directed to the corresponding author.
